# Potential of right ventricular function assessment with echocardiography in transcatheter aortic valve replacement:

**DOI:** 10.1186/s13019-024-03198-5

**Published:** 2024-12-30

**Authors:** Yoshihisa Morita, Taro Kariya, Michael Dougherty, Andrew Peters, Nicholas Ruggiero

**Affiliations:** 1 Department of Anesthesiology, Thomas Jefferson University, 111 South 11th St Gibbon Building, Suite 8280, Philadelphia, PA 19107 USA; 2 Department of Anesthesiology, University of Tokyo, Tokyo, 113-8654 Japan; 3 Department of Cardiology, Thomas Jefferson University, Philadelphia, PA 19107 USA

**Keywords:** Speckle track echocardiography, Transcatheter aortic valve replacement, Atrioventricular block, Right ventricular function

## Abstract

**Background:**

Right ventricular (RV) function assessment by echocardiography can be challenging due to its complex morphology. Also, increasing use of sedation rather than general anesthesia for transfemoral approach transcatheter aortic valve replacement (TAVR) reduces the need for intraoperative transesophageal echocardiography (TEE). Recent clinical studies have demonstrated the importance of 3-dimensional (3D) echocardiography and a longitudinal strain for RV function assessment. In this study, we compared RV function echocardiographic assessment methodologies in TAVR and investigated its clinical utility.

**Methods:**

This was a prospective, observational study of TAVR at a large academic hospital. Inclusion criteria were adult patients undergoing TAVR requiring intraoperative TEE between April 2023 and October 2023. Exclusion criteria include an absolute contraindication to TEE, a pacemaker, or suboptimal intraoperative echocardiography images. The primary goal is to assess the correlation of 3D RV ejection fraction (EF) with RV fraction area change (FAC), and tricuspid annular plane systolic excursion (TAPSE). The secondary goal is to assess the correlation of RV free wall longitudinal strain (FWLS) with any newly diagnosed postoperative ventricular arrhythmia, including complete atrioventricular block (CAVB) and left bundle branch block (LBBB).

**Results:**

Among 33 patients who underwent TAVR, 4 patients were excluded due to poor image quality, and 7 patients were excluded due to existing pacemaker. Thus, data for 22 patients were analyzed in this study. There was a good correlation between 3D RVEF and RV FAC (correlation coefficient 0.789. *p* = 0.0000482), but poor correlation between 3D RVEF and TAPSE (correlation coefficient 0.182. *p* = 0.444). Eight patients developed a new left or right BBB and CAVB postoperatively, and 3 patients required permanent pacemaker. Regression analysis of pre and post valve deployment showed RV free wall RVFWLS was each correlated with postoperative new BBB or CAVB (pre valve deployment: hazard ratio 1.272, 95% CI 1.075 to 1.505, *p* = 0.004981; post valve deployment: hazard ratio 1.134, 95% CI 1.001 to 1.286, *p* = 0.04846). No mortality was reported during the follow-up period, and no significant tricuspid regurgitation (more than moderate) was reported.

**Conclusion:**

3D RVEF and RV FAC showed a good correlation. Intraoperative RVFWLS has the potential to predict postoperative new occurrence of BBB or CAVB.

## Introduction

Transcatheter aortic valve replacement (TAVR), an established treatment for symptomatic aortic stenosis, has advantages over surgical aortic valve replacement (SAVR), such as reduced invasiveness and shorter hospital stays. Right ventricular (RV) function impacts the treatment outcomes after SAVR. However, the effect of RV function on the outcomes of TAVR has shown controversial results [1, 2], which can be attributed to the fact that RV function assessment by echocardiography can be challenging due to its complex morphology and inconsistent methods of quantitative RV function assessment [3]. Actually, there are insufficient studies that compare the RV function echocardiographic assessment methodologies in TAVR. Moreover, the recent trends to switch to monitored anesthesia care sedation from general anesthesia for transfemoral approach decreases the chances of conducting intraoperative transesophageal echocardiography (TEE); intraoperative cardiac function assessment relies on less information [4]. Longitudinal strain (LS) analysis method by speckle tracking echocardiography (STE) directly measures the myocardial deformation of all segments of the RV free wall throughout the whole cardiac cycle and is less angle-dependent than conventional echocardiographic parameters [3]. Recently, the importance of 3-dimensional (3D) echocardiography and potential of STE derived RV free wall LS for RV function assessment have been reported in various clinical studies and is included in the guideline of echocardiographic quantitative chamber assessment [3]. Of note, 3D echocardiography has been extensively validated against cardiac MRI [3]. Recent technological advancements have expedited these assessments with great reproducibility within a short time, although it is still not conducted in real-time. This study aimed at comparing the RV function echocardiographic assessment methodologies in TAVR and discussing their clinical potential in predicting the postoperative course.

## Methods

### Study design

This study was approved by the Institutional Review Board of Thomas Jeferson University (IRB # iRISID-2022-0802) and verbal consent was obtained from all the participants before enrollment per IRB approval. It was registered at ClinicalTrial.gov (NCT 05804240) on August 14, 2023. This study is a pilot prospective observational study of a cohort of adult patients who underwent TAVR (transfemoral or right axillary artery approach) and required intraoperative TEE at an academic institute. At our institute, general anesthesia with TEE is a standard of care for most TAVRs. TEE data were collected from intraoperative TEE images as part of a prospective echocardiographic protocol using 2-dimensional and 3D TEE views. The demographics, perioperative clinical information, and postoperative outcomes of these patients were collected from a computerized patient database.

### Patient cohort

The patient cohort included adult patients undergoing TAVR and requiring intraoperative TEE between April 2023 and October 2023 at Thomas Jefferson University Hospital. The exclusion criteria were: (1) patient refusal to participate in the study; (2) absolute contraindication to TEE; (3) existing pacemaker; and (4) suboptimal echocardiography images. All enrolled patients received general anesthesia with endotracheal intubation, standard American Society of Anesthesiologists monitoring, arterial blood pressure monitoring, and comprehensive TEE examination with a designated protocol. The benefit of real-time RV assessment was that it would be helpful to determine postoperative intervention or disposition. Intraoperative management with anesthetics, mechanical ventilation, vasopressors, inotropic agents, and fluids/transfusions was performed based on department protocols, such as maintaining anesthesia with sevoflurane, remifentanil infusion, a mean arterial pressure > 65 mmHg, tidal volume of 6–8 ml/ideal body weight (kg), and positive end-expiratory pressure of 5–7 cmH_2_O.

### Data collection

After induction of general anesthesia, a 6Vt TEE probe (GE Healthcare, Bensalem, PA, USA) was inserted into the esophagus of each patient. TEE images were intraoperatively collected by the National Board of Echocardiography-certified advanced perioperative echocardiographers who were blinded to the study design using GE Vivid E95 (GE Healthcare, Bensalem, PA); measurements were performed on the TEE machines. The designated views for TEE images were as follows: 4-dimensional (4D) auto right ventricle quantification (RVQ) and automated functional imaging (AFI) on the RV-centered mid-esophageal four-chamber view; 4D RVQ-deprived indices include RV end-diastolic volume, RV end systolic volume, RV ejection fraction (EF), RV stroke volume (SV), RV end-diastolic diameter base, RV diastolic diameter mid, RV longitudinal distance, tricuspid annular plane systolic excursion (TAPSE), and RV fractional area change (FAC) [5]. AFI derived indices include STE derived RV free wall (base, mid, and apical) LS (RVFWLS) [6]. Septum strains were excluded due to the left ventricular effect. The above-mentioned exclusion criteria (3) and (4) is because TEE packages (RVQ and AFI) require good image quality and pacemakers would affect longitudinal strain [5, 6]. Image acquisition was performed twice; the first time was after general anesthesia induction and stabilization of hemodynamics, while the second time was after valve deployment and stabilization of hemodynamics. While acquiring these views, the TEE device setting was not changed and the iSCAN button was pressed before video acquisition.

### Outcomes

The primary outcome was to compare 3D RVEF, RV FAC, and TAPSE. The secondary outcome was to assess the correlation between RVFWLS and any newly diagnosed postoperative conduction disturbance, including complete atrioventricular block (CAVB) and left bundle branch block (LBBB).

### Statistical analyses

Continuous variables with a normal distribution are displayed as the mean ± standard deviation, while variables with a non-normal distribution are displayed as the median and interquartile range. Categorical variables are presented as proportions and absolute numbers. For continuous variables, normality was tested using the Kolmogorov–Smirnov test.

The differences between 2 groups were investigated using Chi-square test or Fisher’s exact test if any of the expected frequencies were < 5 for categorical variables and unpaired and paired Student’s *t*-tests or the Mann–Whitney U test for continuous variables.

The correlation of the two echocardiographic indices was assessed using Pearson’s or Spearman’s rank correlation tests. Univariate Cox Regression analysis was performed for echocardiographic indices and incidence of newly-diagnosed CAVB and LBBB.

Reproducibility of echocardiographic RV indices were assessed using intraclass correlation as follows; two blinded echocardiographers obtained the measurements on the same images for 10 patients who were randomly chosen.

All statistical analyses were performed using R software (version 4.0.2, The R Foundation for Statistical Computing, Vienna, Austria). Statistical significance was set at a p-value < 0.05. All the statistical analyses were performed with EZR (Saitama Medical Center, Jichi Medical University, Saitama, Japan), which is a graphical user interface for R software [6]. More precisely, it is a modified version of R commander designed to add statistical functions that are frequently used in biostatistics [7]. 

## Results

**Fig. 1:**
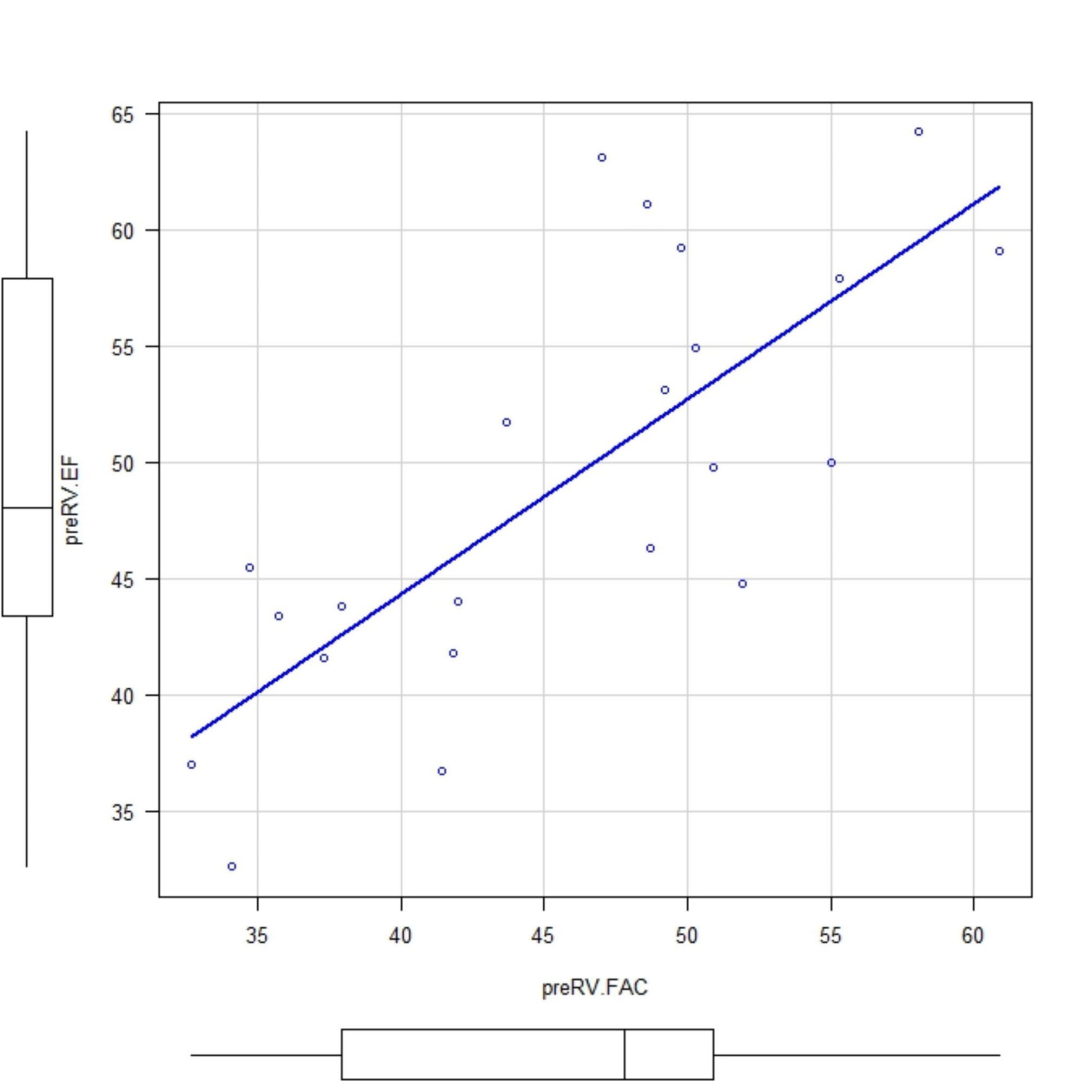
Comparison between preRVEF and preRV FAC. Correlation coefficient: 0.789; *p* = 0.0000482. Abbreviations: preRVEF: pre valve deployment right ventricular ejection fraction, preRV FAC:; pre valve deployment right ventricular fraction area change

**Fig. 2:**
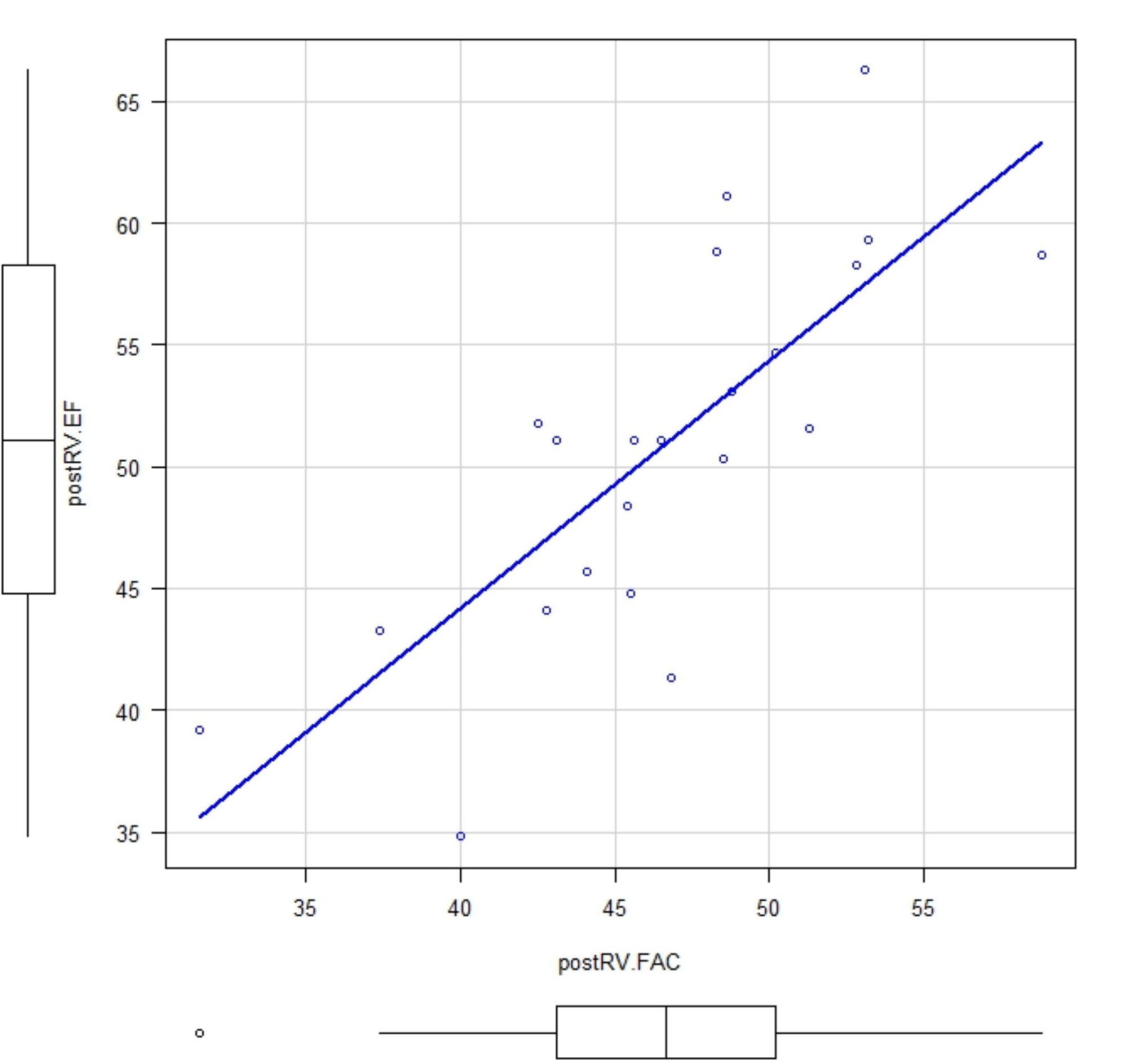
Comparison between postRVEF and postRV FAC. Correlation coefficient: 0.774; *p* = 0.0000388. Abbreviations: postRVEF: post valve deployment right ventricular ejection fraction, postRV FAC:; post valve deployment right ventricular fraction area change

**Fig. 3:**
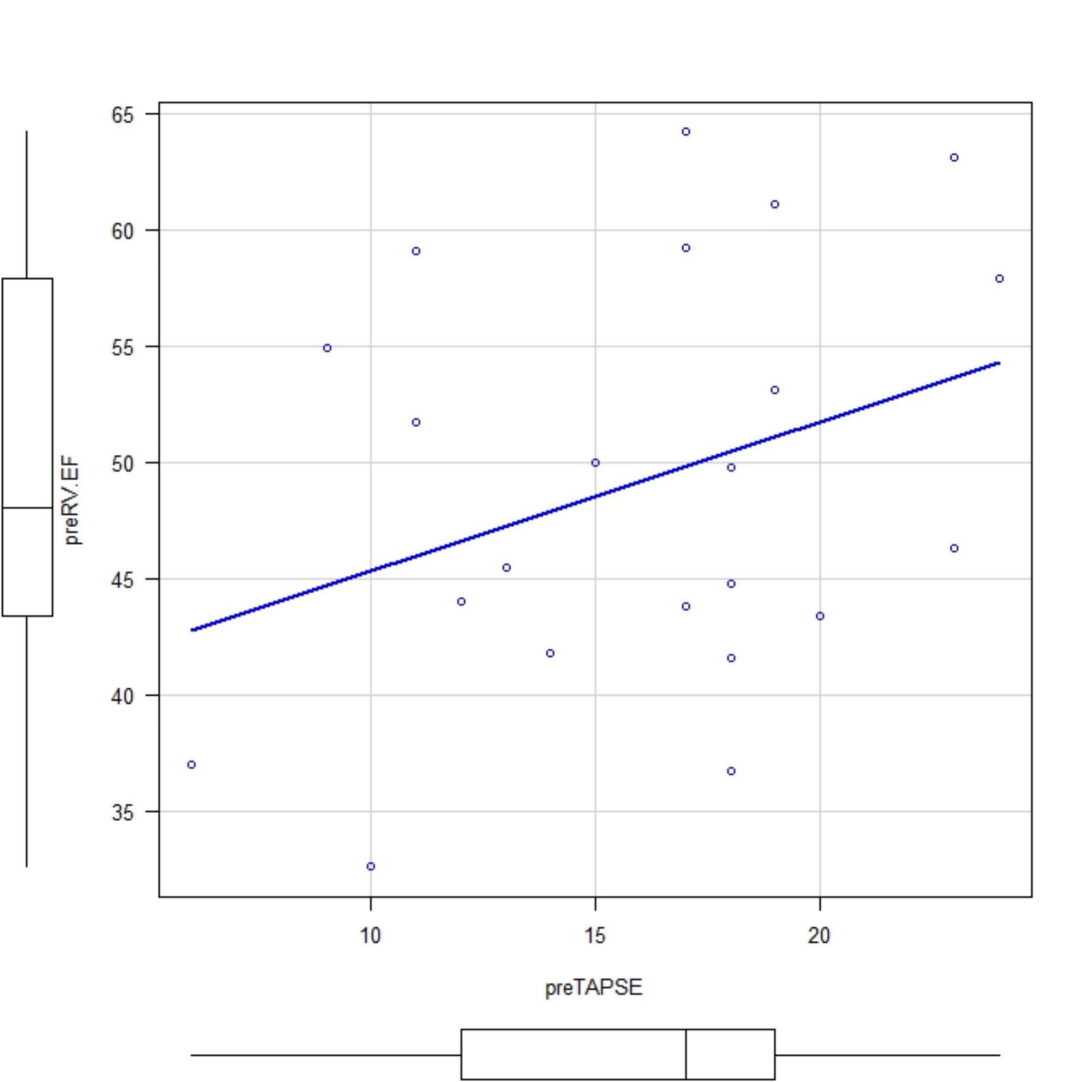
Comparison between preRVEF and preTAPSE. Correlation coefficient: 0.182; *p* = 0.444. Abbreviations: preRVEF: pre valve deployment right ventricular ejection fraction, preTAPSE:; pre valve deployment tricuspid annular plane systolic excursion

**Fig. 4:**
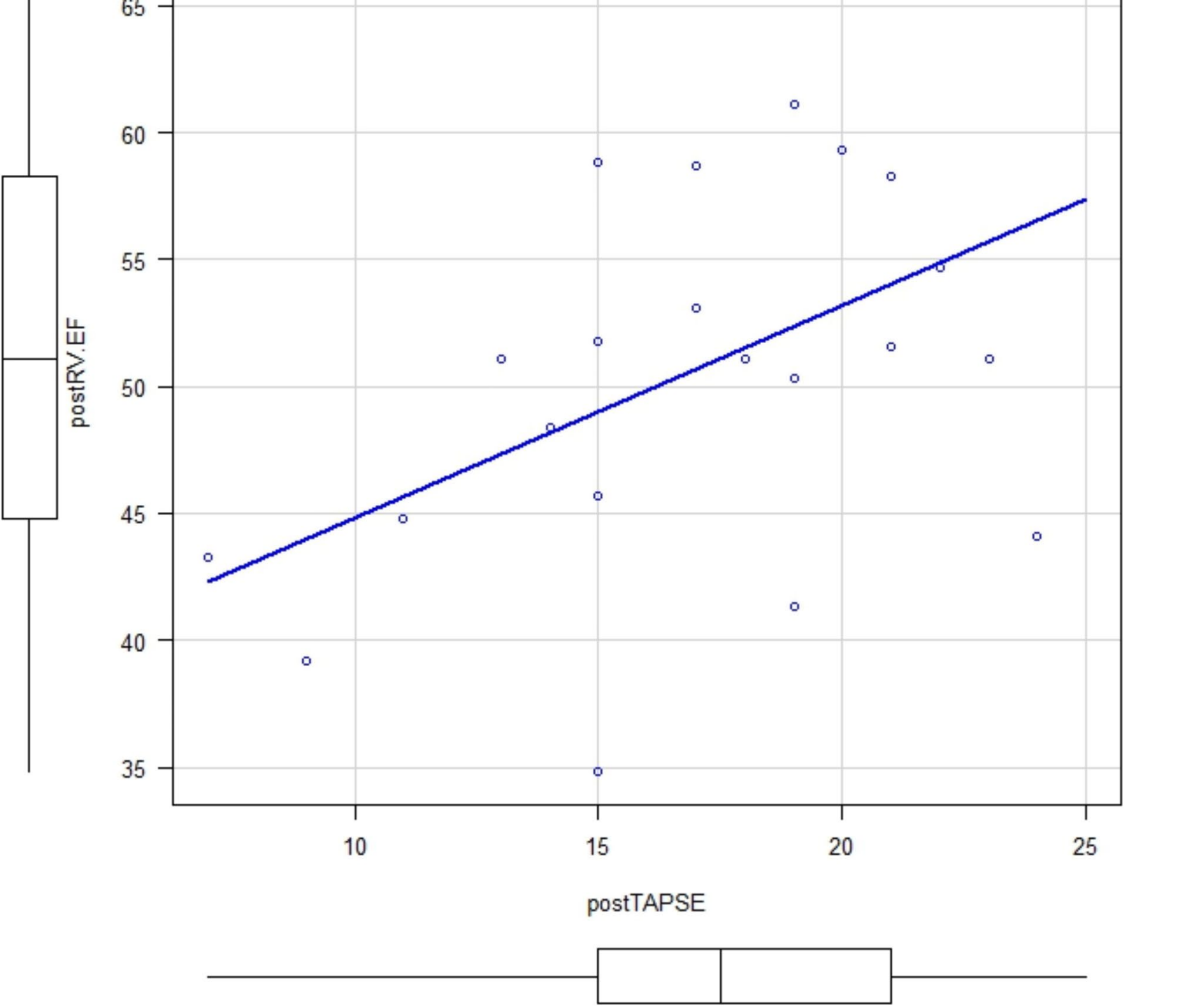
Comparison between postRVEF and postTAPSE. Correlation coefficient: 0.455; *p* = 0.0.83. Abbreviations: postRVEF: post valve deployment right ventricular ejection fraction, postTAPSE:; post valve deployment tricuspid annular plane systolic excursion

Among the 33 patients who underwent TAVR (SAPIEN 3 Ultra valve. Edwards Lifesciences Corp, Irvine, CA) four patients were excluded due to poor image quality and seven patients were excluded due to having an existing pacemaker. Thus, a total of 22 patients were enrolled in this study (Table 1). There was a good correlation between 3D RVEF and RV FAC (pre valve deployment: correlation coefficient, 0.789; *p* = 0.0000482 and post valve deployment: correlation coefficient, 0.774; *p* = 0.0000388), but an inconsistent correlation was observed between 3D RVEF and TAPSE (pre valve deployment: correlation coefficient 0.182; *p* = 0.444 and post valve deployment: correlation coefficient, 0.455; *p* = 0.0383)(Figs. 1, 2, 3 and 4). Postoperatively, four patients developed new LBBB, five patients developed CAVB, and three patients required a permanent pacemaker. The regression analyses of pre- and post-valve deployment are summarized in Table 2. Pre-valve deployment LS in the basal and mid-lateral RV wall was correlated with postoperative new LBBB or CAVB. None of the right ventricular function indices were significantly changed pre/post TAVR (Table 3). No mortality was reported during the follow-up period and no significant tricuspid regurgitation (more than moderate) was reported. Echocardiographic RV function indices in the pre-TAVR and post-TAVR groups are summarized in Table 3. The intraclass correlation for 3D RVEF, RV FAC, and RV free-wall LS showed very strong correlations (> 0.90; Table 4).

**Table 1:** Demographics of patients

	New onset LBBB or CAVB (*N* = 10)	No new onset LBBB or CAVB (*N* = 12)	*p*-value
Males	7	6	0.415
Age (yo)	77.0 [73.00–81.5]	74.5 [68.75–80.0]	0.389
BMI	30.45 [27.33–34.46]	28.92 [27.58–33.17]	0.722
HFpEF	8	8	0.646
AR	2	3	1
MS	0	0	NA
MR	2	0	0.214
TR	0	0	NA
PR	0	0	NA
HTN	10	8	0.214
CAD	5	4	1
Coronary revascularization	3	1	0.293
DM	4	3	0.659
OSA	2	1	0.571
HLD	7	7	1
CKD	2	0	0.214
HD	1	0	0.476
CVA	1	0	0.476
Hypothyroid	2	1	0.553
LVEF (%)	60.00 [55–60.00]	62.5 [50–66.25]	0.44
preTAPSE (mm)	13.5 [9.75–17.25]	18.0 [15.75–19.00]	0.103
postTAPSE (mm)	15.0 [13.0–17.0]	19.0 [16.5–21.25]	0.0322
preRV FAC (%)	36.80 [34.55–41.50]	49.50 [47.36–52.75]	0.00216
postRV FAC (%)	44.10 [40.00–45.60]	48.55 [46.48–51.75]	0.0118
preRV EF (%)	36.925 [42.6–44.225]	52.40 [45.93–59.13]	0.00549
postRV EF (%)	48.40 [43.30–51.10]	53.25 [48.93–58.93]	0.0426
preSBP (mmHg)	119.11 ± 14.56	119.0 ± 15.72	0.987
preDBP (mmHg)	54.4 ± 7.47	54.4 ± 11.57	0.99
preMAP (mmHg)	76.0 ± 6.48	76.0 ± 12.2	1
preHR (BPM)	62.33 ± 11.81	61.0 ± 8.29	0.77
postSBP (mmHg)	134.77 ± 21.4	128.6 ± 17.6	0.499
postDBP (mmHg)	59.86 ± 11.3	58.4 ± 13.9	0.846
postMAP (mmHg)	84.63 ± 11.86	81.8 ± 14.24	0.646
postHR (BPM)	73.0 ± 19.2	63.2 ± 9.04	0.166
Crystalloid (ml)	1100 [1000–1400]	850 [600–1000]	0.161
On inotropes/vaspressors	2	1	0.553
Valve size, 20 mm	0	1	
Valve size, 23 mm	3	3	
Valve size, 26 mm	6	6	
Valve size, 29 mm	1	2	

The data are presented as median [IQR]. LBBB, left bundle branch block; CAVB, complete atrioventricular block; BMI, body mass index; HFpEF, heart failure with preserved ejection fraction; AS, aortic stenosis; AR, aortic regurgitation; MS, mitral stenosis; MR, mitral regurgitation; TR, tricuspid regurgitation; PR, pulmonary regurgitation; HTN, hypertension; CAD, coronary artery disease; DM, diabetes mellitus; OSA, obstructive sleep apnea; HLD, hyperlipidemia; CKD, chronic kidney disease; HD, hemodialysis; CVA, cerebrovascular accident; LVEF, left ventricular ejection fraction; preTAPSE, pre valve deployment tricuspid annular plane systolic excursion; postTAPSE, post valve deployment tricuspid annular plane systolic excursion; preRV FAC, pre valve deployment right ventricular fraction area change; postRV FAC, post valve deployment right ventricular fraction area change; preRV EF, pre valve deployment right ventricular ejection fraction; post RV EF, post valve deployment right ventricular ejection fraction; preSBP, pre valve deployment systolic blood pressure; preDBP, pre valve deployment diastolic blood pressure; preMAP, pre valve deployment mean arterial pressure; preHR, pre valve deployment heart rate; postSBP, post valve deployment systolic blood pressure; postDBP, post valve deployment diastolic blood pressure; postMAP, post valve deployment mean arterial pressure; postHR, post valve deployment heart rate

**Table 2:** Comparison of correlation of STE, 3D RVEF, and RV FAC with a new onset LBBB or CAVB

Pre TAVR	Post TAVR
Basal RV LS	Basal RV LS
1.122 [1.041–1.21], *p* = 0.00276	1.015 [0.945–1.09], *p* = 0.680
Mid RV LS	Mid RV LS
1.083 [1.009–1.162], *p* = 0.0268	1.049 [0.984–1.118], *p* = 0.14
Apical RV LS	Apical RV LS
1.054 [0.983–1.131], *p* = 0.139	0.972 [0.905–1.044] *p* = 0.437
3D RVEF	3D RVEF
0.8753 [ 0.770–0.995], *p* = 0.0415	0.909 [0.8112–1.018], *p* = 0.0974
RV FAC	RV FAC
0.8419 [0.731–0.970], *p* = 0.0175	0.838 [0.736–0.954], *p* = 0.00753

TAVR, transcatheter aortic valve replacement; RV LS, right ventricle longitudinal strain; 3D RVEF, three-dimensional right ventricular ejection fraction; RV FAC, right ventricular fraction area change

**Table 3:** Right ventricular function indices in pre/post TAVR

	PreTAVR	PostTAVR	*p*- value
TAPSE	15.70 ± 4.747	17.048 ± 4.759	0.0583
RV FAC	45.250 ± 8.314	46.290 ± 5.872	0.321
RV SV	46.150 ± 19.930	48.190 ± 19.518	0.247
3D RV EF	48.425 ± 8.906	50.504 ± 7.796	0.0803
RV free wall LS	-26.333 ± 7.839	-28.491 ± 390	0.186

TAVR, transcatheter aortic valve replacement; RV FAC, right ventricular fraction area change; RV SV, right ventricular stroke volume; 3D RVEF, three-dimensional right ventricular ejection fraction; RV LS, right ventricle longitudinal strain; LS.S., LS adjusted with systolic blood pressure (LS.S.= LS x systolic blood pressure/120 mmHg); LS.D., LS adjusted with diastolic blood pressure (LS.D.= LS x diastolic blood pressure/80 mmHg

**Table 4:** Intraclass correlation analysis for 3D RVEF, RV FAC, TAPSE, and RVFWLS

	Intraclass Correlation	95% Confidence Interval		F Test with True Value 0			
		Lower Bound	Upper Bound	Value	df1	df2	*p*-value
(A) **RV FAC**							
Single Measures	0.921	0.722	0.979	22.749	9	9	< 0.001
Average Measures	0.959	0.839	0.990	22.749	9	9	< 0.001
(B) **TAPSE**							
Single Measures	0.840	0.504	0.957	11.469	9	9	< 0.001
Average Measures	0.913	0.670	0.978	11.469	9	9	< 0.001
(C) **TAPSE**							
Single Measures	0.840	0.504	0.957	11.469	9	9	< 0.001
Average Measures	0.913	0.670	0.978	11.469	9	9	< 0.001
(D) **RVFWLS**							
Single Measures	0.957	0.652	0.991	81.444	9	9	< 0.001
Average Measures	0.978	0.789	0.995	81.444	9	9	< 0.001

3D RVEF, three-dimensional right ventricular ejection fraction; RV FAC, right ventricular fraction area change; TAPSE, tricuspid annular plane systolic excursion; RVFWLS, RV free-wall longitudinal strain

## Discussion

Due to technological advances and better familiarity with TAVR, a major reduction in perioperative complications has been noted [4, 8–10]. However, TAVR-related conduction disturbances, mainly new-onset LBBB and CAVB requiring permanent pacemaker implantation, remain the most common complications of this procedure [11]. These complications have been associated with several factors such as prior right BBB, transcatheter valve type, and implantation depth [11–14]. New-onset LBBB and the need for permanent pacemaker implantation may have a significant detrimental association with the prognosis of patients, and early risk stratification is crucial to achieve a timely intervention.

Our study has two main findings; first, there was a good correlation between 3D RVEF and RV FAC, but not with TAPSE, second, the baseline RVFWLS, excluding the apex, correlated with the occurrence of newly diagnosed LBBB or CAVB.

### 3D RVEF, RV FAC, and TAPSE in TAVR

The 4D RVQ package provides RV size, TAPSE, RV FAC, and 3D RVEF with a single measurement [5]. Also, 4D RVQ package is applicable to TEE and the TAPSE measurement can be accurate as long as there is an acceptable image quality, number of 4D frames, and reference points well aligned [5]. 

The 3D RVEF has a good correlation with cardiac magnetic resonance-measured RVEF, which is the gold standard for RV systolic function assessment [5, 15]. The 3D RVEF reflects longitudinal and radial components of RV systole, which overcomes the geometric limitations of traditional echocardiographic parameters for evaluating the RV function [3]. The 3D RVEF can explore the entire right chamber, including the RV outflow tract, which can include the contribution of the RV outflow tract to the global systolic function [3]. However, it cannot directly represent systolic performance because it is load dependent [3]. The RV FAC reflects longitudinal and radial information of the RV systole, which overcomes the shortcoming that it is only confined to a single motion type [3]. It correlates with RVEF derived from cardiac magnetic resonance and can provide prognostic information in patients with HF [3, 16]. However, RV FAC has several drawbacks that limit its clinical application. It depends on load and ignores the effect of the RV outflow tract on ejection; [3] the endocardial delineation also needs superior image quality. Another main limitation of RV FAC is that the obscure definition of the RV lateral wall results in inconsistent interobserver reproducibility [3]. Our study utilized echocardiography with improved artificial intelligence technology [5], which enabled us to overcome the challenge of RV lateral wall definition and achieved very good reproducibility (Table 4). As previously reported, our study noted a good correlation between RVEF and RV FAC [3], but not between RVEF and TAPSE. TAPSE has been included in RV systolic function indices because RV contraction mainly consists of longitudinal movement of RV wall, but TAPSE might not be an accurate surrogate of RV systolic function in RV regional dysfunction (particularly in septal dysfunction) or inconsistent measurement angle [3]. The TAPSE measured in this study was obtained by employing the 4D RVQ function using the same image that was used for RVEF. Thus, TAPSE in our study was more angle-independent than that of the 2-dimensional assessment [5]. In addition, TAPSE is afterload dependent (i.e., pulmonary artery pressure) and shows a decrease with increasing pulmonary artery pressure when the apex of the heart is often pulled toward the LV during systole. Accordingly, the tricuspid annulus is pulled along in unison, even in the absence of actual shortening or deformation of the RV [17, 18]. This apex “rocking” in high pulmonary artery pressure would compromise the utility of TAPSE. In this study, we did not measure pulmonary artery pressure at the time of our assessment, but given that our patient population included 3 obstructive sleep apnea patients, obstructive sleep apnea might be also contributing to increased RV afterload. This inconsistency in correlation of each RV function index emphasizes the importance of comprehensive assessment of RV function [18]. 

### Potential of RVFWLS to predict postoperative CAVB and LBBB

In TAVR, most conduction disturbances occur in the acute period (periprocedural or within 24.

hours of the procedure) [19, 20], and new-onset LBBB has been reported more frequently when the self-expandable CoreValve system (Medtronic Inc, Minneapolis, MN) is used than that when the balloon-expandable Edwards SAPIEN/SAPIEN XT valve (Edwards Lifesciences LLC, Irvine, CA) or Edwards SAPIEN 3 valve (Edwards Lifesciences LLC, Irvine, CA; 18–65% vs. 4–30% vs. 12–22%, respectively) is used [11, 21–25]. Although there is inconsistent data regarding correlation of a new onset LBBB and mortality, LBBB should be considered given that it can eventually lead to CAVB requiring permanent pacemaker as reported in 13% of the patients in a study including 45% self-expandable valves recipients compared to 8% of the patients implanted with a balloon-expandable valve [11]. The mechanism of the new onset LBBB can be multifactorial, but it has been hypothesized that baseline subclinical conduction abnormality can be more clinically apparent with constant compression of the conduction system by the transcatheter valve [11]. 

On the other hand, STE is validated for assessing RV strain and included in the most updated echocardiographic RV function assessment guidelines and has been investigated in terms of its clinical implications [3, 16, 26]. STE derived LS can detect early signs of deterioration of cardiac function and can be useful for predicting future mortality [16]. The advantages of STE include its angle independence and great reproducibility [3], while its disadvantages include being vendor-dependent and affected by afterload [3]. AFI package used in our study is validated for assessing RV strain [6]. 

In our study, pre valve deployment LS in basal and mid lateral RV wall, but not apical RV wall LS, were correlated with the new onset of LBBB or CAVB. This can be due to the fact that the apical RV wall is far away from the new aortic valve, and impaired LS of the basal-mid RV wall might represent a vulnerable conductive pathway in the basal-mid septum where bundle branches run. Imai et al. reported that RV molecular changes might occur in earlier stages of left ventricular failure before RV hemodynamic changes are noted [27]. Also, in patients with severe AS, chronic pressure overload in the LV chambers can be transmitted through the pulmonary vasculatures and result in compensatory RV remodeling, dilatation, and eventually RV dysfunction which is more purely detected in basal-mid free wall and might be an indication of more impaired LV [28]. Pibarot et al. emphasized the importance of RV function assessment during perioperative management for patients with TAVR [29]. With the LS potential to detect early signs of cardiac failure, RVFWLS in our study might have detected subclinical RV molecular changes.

When acquiring images for STE assessment, it is very important to angle the images so that calcification of the valve does not interfere with the RV free wall. STE itself is not angle dependent; thus, we were able to acquire the images by simple TEE probe manipulation in 29 out of the 33 cases (87.9%). It is also important to use an RV-centered 4 chamber view with a frame rate greater than 50 to achieve a high-quality image.

## Limitations

Our study had some limitations. First, it was a single-center study with a small number of patients. Second, our follow-up period was relatively short (within 6 months) and may not be long enough for observing all the postoperative complications, though previous studies have reported that 60–96% of post TAVR cases of high-degree AVB occur within 24 h postoperatively [11, 30]. 

Third, there is a potential effect of general anesthesia on TEE assessment of RV function. Given that the recent trend is to perform transfemoral TAVR under sedation unless contraindicated, caution should be taken to translate this study to clinical practice. In addition, there were no reported cases of mortality, and we could not assess the correlation between echocardiographic RV function indices and mortality. In future studies, a larger number of patients and a longer follow-up period are needed.

## Conclusion

In TAVR, 3D RVEF should be used for RV function assessment. In addition, RVFWLS might be able to predict new-onset LBBB or CAVB. Further studies which assess long term outcome are required to confirm this hypothesis.

## Data Availability

All data generated or analysed during this study are included in this published article.

## Abbreviations

3D: 3 Dimensional
CAVB: Complete atrioventricular block
FAC: Fractional area change
LBBB: Left bundle branch block
LS: Longitudinal strain
RV: Right ventricle
RVQ: Right ventricle quantification
SAVR: Surgical aortic valve replacement
STE: Speckle tracking echocardiography
RVFWLS: Right ventricle free wall longitudinal strain
SV: Stroke volume
TAPSE: Tricuspid annular plane systolic excursion
TAVR: Transcatheter aortic valve replacement
TEE: Transesophageal echocardiography
